# Advancing Clinical Judgment Through Innovation and Lifelong Learning

**DOI:** 10.51894/001c.169277

**Published:** 2026-09-03

**Authors:** Francis Akenami, Rana Ismail, Andrea Amalfitano

**Affiliations:** 1 College of Osteopathic Medicine – Research, Innovation, and Scholarly Engagement (RISE) Michigan State University, East Lansing, MI, USA; 2 College of Osteopathic Medicine – Graduate Medical Education Alliance Michigan State University, East Lansing, MI, USA

**Keywords:** clinical reasoning, medical education, patient safety, post-marketing surveillance, surgical innovation, diagnostic imaging

## INTRODUCTION

The articles in Volume 11, Issue 1 of the Spartan Medical Research Journal illustrate a central obligation of contemporary medicine: to act decisively while remaining alert to what is not yet apparent. Across emergency care, infectious diseases, cardiovascular medicine, surgery, orthopedics, and medical education, this issue shows how careful observation, disciplined use of evidence, and continual learning can transform uncertainty into safer, more effective care.

The issue also reflects the Journal’s commitment to scholarship that is clinically useful. Its contributions do not merely describe uncommon presentations or emerging interventions; they clarify the questions clinicians, educators, and health systems should ask when familiar assumptions are insufficient.

## DIAGNOSTIC HUMILITY AND THE VALUE OF RECONSIDERATION

Several reports underscore that an initially reassuring or seemingly obvious presentation can conceal substantial risk. Evans and colleagues describe a high-velocity collision in which a patient with initially stable hemodynamics had a rare superior mesenteric artery branch injury. Early imaging, close observation, and timely operative management were decisive in preventing further deterioration.^1^ The report is a reminder that the mechanism of injury and evolving clinical picture must remain integral to trauma assessment.

Vierra and colleagues similarly demonstrate the danger of diagnostic momentum. Their case of HSV-2 proctitis with erythema multiforme closely resembled secondary syphilis, yet targeted polymerase chain reaction testing established the correct diagnosis and enabled appropriate antiviral treatment.^2^ The lesson extends beyond sexually transmitted infections: when clinical features do not fully cohere, clinicians should revisit the differential diagnosis and use confirmatory testing thoughtfully.

Esmaili and colleagues add a further dimension to this theme through their report of prosthetic valve endocarditis caused by an organism within the Mycobacterium smegmatis group. Persistent bacteremia and symptoms were not explained by serial transesophageal echocardiography; 18F-FDG PET/CT provided the critical evidence of prosthetic infection and supported surgical source control.^3^ Their case reinforces the importance of multidisciplinary collaboration and of escalating diagnostic strategies when standard studies are non-diagnostic but clinical concern persists.

## INNOVATION REQUIRES SURVEILLANCE AND SURGICAL PRECISION

The issue also considers innovation through the lens of safety. Gopannagari and colleagues analyze post-marketing reports of renal denervation for uncontrolled hypertension in the FDA MAUDE database. Their findings identify vascular and device-related events that, although uncommon and not sufficient to establish incidence or causality, warrant continued vigilance as use expands.^4^ The study exemplifies the complementary role of real-world surveillance alongside randomized trials.

In orthopedics, Mudiganty and colleagues review contemporary trochleoplasty for recurrent patellofemoral instability associated with high-grade trochlear dysplasia.^5^ By synthesizing indications, techniques, outcomes, and complications, the review helps clinicians match a technically demanding intervention to appropriately selected patients. Baghdadi and colleagues provide a surgical counterpart: their report of a duplicated vas deferens identified during laparoscopic herniorrhaphy illustrates how meticulous anatomic identification protects patients from preventable injury.^6^ Together, these articles affirm that innovation achieves its value only when accompanied by sound selection, technical discipline, and respect for individual anatomy.

## LEARNING SYSTEMS FOR A CHANGING CLINICAL ENVIRONMENT

Clinical excellence depends on learning systems that fit the needs of practicing faculty and trainees. Figg and colleagues compare an interactive e-learning module with a text-based resource for faculty development. While quantitative comparisons were exploratory and underpowered, participants valued the e-learning module for learning and the text resource for later reference; the combined approach was preferred.^7^ This practical insight is especially relevant as medical education increasingly combines asynchronous learning, interactive design, and accessible point-of-need resources.

Taken together, the issue encourages a broad view of quality: it is not only the correct diagnosis or a successful procedure, but also the habits and systems that make those outcomes more likely. These include reassessing assumptions, integrating new diagnostic tools judiciously, monitoring interventions after adoption, preserving technical precision, and designing education for real clinical use.

## CONCLUSION

Volume 11, Issue 1 presents scholarship grounded in the realities of patient care: rare but consequential findings, complex diagnostic pathways, evolving interventions, and the ongoing work of professional development. We are grateful to the authors, reviewers, and editorial contributors whose efforts make this dialogue possible. We hope these articles prompt readers not only to apply the findings in their own settings, but also to remain curious, careful, and prepared to look again when the clinical story demands it.
